# The Comparative Value of Feline Virology Research: Can Findings from the Feline Lentiviral Vaccine Be Translated to Humans?

**DOI:** 10.3390/vetsci4010007

**Published:** 2017-01-28

**Authors:** Margaret J. Hosie, Navapon Techakriengkrai, Paweł M. Bęczkowski, Matthew Harris, Nicola Logan, Brian J. Willett

**Affiliations:** 1 MRC-University of Glasgow Centre for Virus Research, Glasgow G61 1QH, UK; pawel.beczkowski@gmail.com (P.M.B.); m.harris.2@research.gla.ac.uk (M.H.); nicola.logan@glasgow.ac.uk (N.L.); Brian.Willett@glasgow.ac.uk (B.J.W.); 2 Department of Veterinary Microbiology, Faculty of Veterinary Science, Chulalongkorn University, Bangkok 10330, Thailand

**Keywords:** feline immunodeficiency virus, human immunodeficiency virus, vaccine, tropism, comparative study

## Abstract

Feline immunodeficiency virus (FIV) is a lentivirus of domestic cats that shares several similarities with its human counterpart, human immunodeficiency virus (HIV). Their analogies include genomic organization, lymphocyte tropism, viral persistence and induction of immunodeficiency. FIV is the only lentivirus for which a commercial vaccine is registered for prevention in either human or veterinary medicine. This provides a unique opportunity to investigate the mechanisms of protection induced by lentivirus vaccines at the population level and might contribute to the development of efficacious HIV vaccines. As well as having comparative value for vaccine studies, FIV research has shed some light on the relationship between lentiviral tropism and pathogenesis. Recent studies in our laboratory demonstrated that the interaction between FIV and its primary receptor changes as disease progresses, reminiscent of the receptor switch observed as disease progresses in HIV infected individuals. Here we summarise findings illustrating that, in addition to its veterinary significance, FIV has comparative value, providing a useful model to explore lentivirus–host interactions and to examine potential immune correlates of protection against HIV infection.

## 1. Introduction

The feline lentiviruses occur worldwide and are important pathogens of domestic cats and endangered felids alike. As the sole non-primate lentivirus that causes immunodeficiency in its natural host species, feline immunodeficiency virus (FIV) has an important role as an animal model for human immunodeficiency virus/acquired immunodeficiency syndrome (HIV/AIDS), a global epidemic affecting an estimated 39 million people [1,2,3]. It is remarkable that the only lentiviral vaccine to have been developed and subsequently marketed across human and veterinary medicine is the vaccine for FIV. Surely it is therefore a priority to study the protective immune responses induced by this efficacious vaccine against naturally occurring lentivirus? Indeed, comparative feline vaccine research offers unique opportunities to inform strategies for the development of comparable vaccines against related pathogens of humans and other animals. Here, we discuss our recent findings that describe the interaction between FIV and its host cellular receptors, comparing the observed switch in receptor usage to the R5-X4 switch reported for the human lentivirus, HIV. We propose that the switch in receptor usage is a consequence and not the cause of disease progression and we suggest that our improved understanding of the switch in virus–receptor interaction, which occurs with disease progression, has implications for lentivirus vaccine design.

## 2. A Commercial FIV Vaccine

From the data obtained from trials of potential FIV vaccine candidates, the current understanding of FIV biology has been extended and potential immune correlates of protection have been examined. The most successful vaccines have been based on whole inactivated virus and/or fixed infected-cells [4,5,6] and indeed, commercially available dual-subtype FIV vaccine contains whole inactivated virus [7]. In 2002, the Fel-O-Vax FIV vaccine (Boehringer-Ingelheim, Ingelheim, Germany) first became available in the USA [8] and it was subsequently released in Canada in 2003, in Australia and New Zealand in 2004 and subsequently in Japan in 2008.

The Fel-O-Vax FIV vaccine has been shown to induce 80% protection against experimental [7,9] and contact challenge [10], as well as protecting cats from infection with heterologous strains [10,11,12]. Given the similarities between FIV infection of cats and HIV infection of humans, a broader understanding of the mechanisms of immunity to infection with FIV has potential to inform the development of candidate HIV vaccines. Given the challenges in developing an effective HIV vaccine [13], the insights offered by comparative studies of lentiviral immunity in other species have significant comparative value. Many experimental HIV vaccine candidates have been tested, with trials resulting sometimes in protection but also in enhanced infection on occasion [13]. Following testing in non-human primate models, four HIV vaccines tested in phase IIb or III efficacy trials in human volunteers [14,15]. These vaccine candidates included VaxGen gp120 (B/B′ and B/E) that was tested in the USA [16,17] and Thailand [18], the Merck Ad5-HIV-1 vaccine that was tested in the STEP trial [19,20] and ALVAC-HIV prime with a recombinant glycoprotein 120 boost that was tested in the RV144 study [21]. The STEP trial was halted early because vaccination increased the risk of volunteers becoming infected with HIV [22], which represented a major setback for HIV vaccine development. However, 30% protection was observed in the RV144 study, which tested four priming injections of a recombinant canarypox vector vaccine (ALVAC-HIV [vCP1521] (Sanofi Pasteur, Lyon, France) followed by two booster injections of recombinant glycoprotein 120 subunit (the AIDSVAX B/E vaccine) in healthy volunteers in Thailand at risk of heterosexual transmission of HIV [21]. Consequently, HIV vaccine efforts are now being focused on prime-boost strategies, although it is likely that an improved understanding of lentiviral biology and the identification of immune correlates of protection will be required before safe and effective human lentiviral vaccines can be developed.

## 3. Why Do Some Virus Strains Resist Vaccine-Induced Protection?

While it is remarkable that a successful feline lentiviral vaccine has been used in cats since 2002, if we are to translate this successful lentiviral vaccine to other species, it is essential that we understand the mechanisms of protection. Experimentally, it has been shown that protection with the Fel-O-Vax FIV vaccine did not extend to experimental challenge with the virulent primary GL8 isolate [23]. However, since the natural challenge dose in FIV infection remains undefined, it is possible that the challenge dose used in experimental studies might be too stringent, such that increased efficacy against heterologous challenge might be observed in cats naturally exposed to FIV infection. Therefore, it will be important to monitor the commercial vaccine’s efficacy under field conditions.

We reported previously [24] the case of a cat that had been vaccinated against FIV annually for three years or more and then died following the diagnosis of FIV infection, three months after the last vaccination. Full-length FIV *env* sequences were cloned from blood and sequence analysis revealed that the cat had been infected with an isolate of FIV containing a recombinant clade A/B FIV envelope glycoprotein. It was striking that the major parent Env shared a high degree of sequence homology with the Env of GL8. Recently, similar case of vaccinated cats that subsequently became infected was reported in the Australian field study [25]. In this study all of the “vaccine-breakthroughs” were infected with GL8 and related subtype A strains. Thus, the findings that GL8 and naturally occurring GL8-related viruses resist vaccine induced protection presented an opportunity to investigate the relationship between receptor usage phenotype and resistance to vaccine-induced protection.

## 4. A Switch in Receptor Usage Phenotype Occurs with Time Post Infection

We have made significant contributions to understanding the early stages of the host-lentivirus interaction identifying CD134 (OX40) and CXCR4 (CD184) as the primary and secondary receptors respectively for FIV [26,27]. It is striking that, in spite of using different primary receptor molecules for host cell entry, FIV and HIV both target CD4^+^ T cells and induce progressive immune dysfunction [28,29]. HIV tropism is governed by the restricted expression patterns of its primary receptor (CD4) and major co-receptors (CXCR4 and CCR5), whereas FIV targets activated CD4^+^ T cells via an initial interaction with CD134 and subsequent interaction with CXCR4. CXCR4-dependent HIV variants accumulate in patients with disease progression, whereas CCR5-dependent variants dominate in early infection [30,31]. It is assumed that the acquisition of CXCR4-usage is a key event in the progression to human AIDS; however, our FIV studies caution against such interpretations, because all FIVs utilise CXCR4 as the sole co-receptor for infection. With disease progression, it was demonstrated that the late variants emerging displayed different modes of interaction with CD134, analogous to late CXCR4-dependent HIV variants.

## 5. Different Modes of Interaction with CD134; CRD2-Dependent and -Independent Isolates

**Figure 1:**
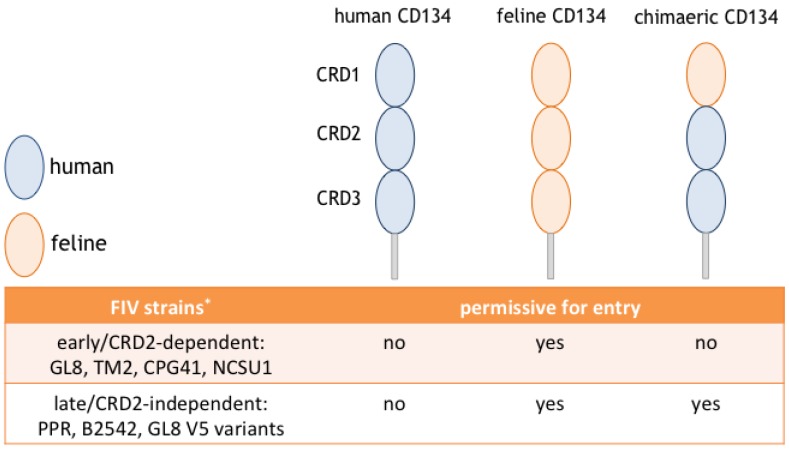
Schematic representations of human, feline and chimaeric CD134 molecules. Oval shapes represent cysteine rich domains (CRD) 1 to 3. Blue and orange ovals represent domains of human and feline CD134, respectively. Cell lines expressing different recombinant molecules of CD134; human CD134, feline CD134 and a chimaeric feline/human CD134 containing only the first CRD of feline CD134, were used to determine the CRD2 dependence of FIV strains. * This table summarises previously reported results [33,34,35].

In common with other members of the tumor necrosis factor receptor superfamily, the feline CD134 molecule contains three cysteine rich domains (CRD 1-3) [32,33]. The receptor-binding site of FIV Env was first mapped to the CRD1 region of the CD134 molecule, in studies using the PPR strain of FIV [34]. However, later studies reported that some strains of FIV require additional determinants on CRD2 for infection [33,34]. Therefore we proposed that FIV strains could be classified, according to their receptor phenotype, as either CRD2-dependent or CRD2-independent (Figure 1). We proposed that binding additional determinants on CRD2 might provide CRD2-dependent strains with more efficient, high affinity interactions with CD134, as CRD2-dependent isolates have been shown to be more resistant to antagonism by anti-CD134 and soluble CD134 ligand [35,36,37].

## 6. CRD2-Independent Variants Emerge in Cats Infected both Experimentally and Naturally with FIV

Initially, we observed that CRD2-dependent strains of FIV (GL8 and CPG41) were isolated during the early phase of infection [35,36,37,38,39,40,41], whereas CRD2-independent strains had either become lab-adapted following extensive in vitro passage or had emerged after several years of infection (PPR and B2542) (Figure 1). Subsequently, following long term monitoring of cats that had been infected experimentally with the GL8 molecular clone of FIV, we detected a quasispecies comprising variants with differing amino acid compositions, neutralisation sensitivities and receptor phenotypes and observed the emergence of CRD2-independent GL8 variants [35,42].

**Figure 2:**
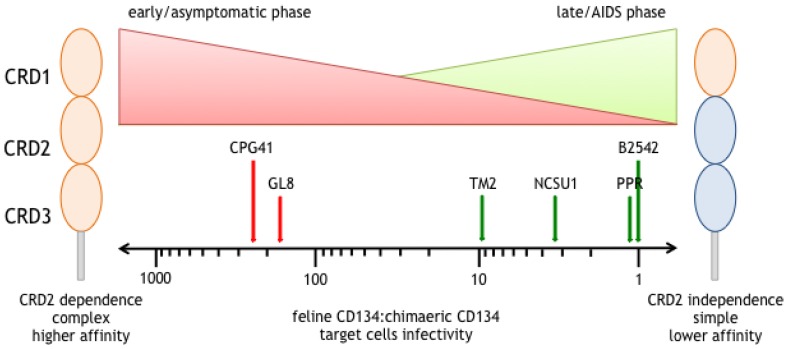
Shift towards CRD2-independent interaction over the course of FIV infection, similar to the switch from R5 to R5X4 or X4 viruses in HIV infection. Oval shapes represent cysteine rich domain (CRD) 1 to 3 of the CD134 molecule; orange and blue represents domains of feline and human CD134 molecule, respectively. CRD2 dependence of FIV isolates was determined by comparing their infectivity on target cells expressing feline CD134 (left, all orange) or chimaeric feline/human CD134 molecule (right, feline CRD1 only). The X-axis shows the infectivity ratios of isolates for the two target cell lines. CRD2 dependent isolates such as CPG41 and GL8 cannot infect target cells expressing the chimaeric receptor, leading to a higher ratio as indicated by the red arrows. In contrast, the CRD2 independent strains PRR and B2542 are equally infectious for both cell lines, having a very low ratio, as indicated by the green arrows. The red and green triangles at the top of the figure depict the theoretically quantities of CRD2-dependent and CRD2-independent variants throughout the clinical course of FIV infection. This figure is summarized from [33,34,43,44,45].

In order to determine whether these experimental findings also extended to naturally infected cats, we analysed FIV isolates from individual, naturally infected cats at sequential intervals, comparing the receptor phenotypes of the viral quasispecies that evolved in cats that remained healthy with those of cats in which disease progression had occurred [43]. We demonstrated that the emergence of CRD2-independent strains of FIV coincides with disease progression (clinically ill cats with either CD4^+^ T cell counts <350 cells/µL or that died). These findings led to the hypothesis that disease progression is associated with viral evolution in which the receptor phenotype of dominant Env variants switches towards CRD2-independence during the course of FIV infection (Figure 2).

## 7. CRD2-Independent Variants Are a Consequence, Not the Cause of Immunodeficiency

This hypothesis was supported by the findings from a study conducted in cats experimentally inoculated with reconstituted quasispecies composed of GL8 and variants containing CRD2-dependent or CRD2-independent Envs [44]. Throughout the 21-week period of study, the variants of GL8 containing CRD2-dependent Envs replicated faster and selectively expanded in all cats. It was demonstrated that the failure to replicate by the CRD2-independent variants was not associated with either the presence of neutralising antibodies or cell-mediated immune responses [44], suggesting that the switch in receptor phenotype also led to decreased replicative fitness.

It has been widely assumed that the switch in receptor usage from CCR5 to CXCR4 is a key event in progression to human AIDS [31,46]. However, we conclude that the accumulation of CRD2-independent strains in feline AIDS (as well as, potentially, CXCR4-dependent viruses in AIDS and viral variants with broader cell tropisms in lentiviral infections per se) is a consequence rather than a cause of immunodeficiency, promoting viral dissemination to novel tissue compartments [45]. This issue is profoundly important when considering strategies for controlling lentiviral infections, since vaccines are required that induce protective immunity against isolates with the receptor phenotype associated with high levels of replication in early infection, i.e., CRD2-dependent FIV variants and CCR5-dependent HIV variants.

## 8. CRD2-Dependent Variants Resist Vaccine Protection

Therefore it appears likely that the resistance of GL8 to vaccine protection is related to its CRD2-dependent receptor phenotype. Similarly, the Env variants from the vaccinated cat that was diagnosed with FIV infection displayed the CRD2-dependent receptor phenotype [33,43]. This finding indicated that the Env variants isolated from the infected vaccinate had been transmitted recently, consistent with the hypothesis that virus isolates from early infection displaying the CRD2-dependent phenotype might be resistant to vaccine-induced protection. Therefore it is important for the future development of lentiviral vaccines that we continue to investigate the mechanisms associated with vaccine protection against FIV infection. Such studies will contribute to the development of the next generation of improved FIV vaccines, in order to protect cats against infection with diverse, pathogenic strains displaying the CRD2-dependent phenotype.

## 9. Enhancement of Lentiviral Infection Following Vaccination

As described previously, the outcomes following the challenge of vaccinates may be efficacious, neutral or in extreme circumstances deleterious, as in the STEP trial testing the Merck Ad5-HIV-1 vaccine [14]. The enhancement of HIV infection that was observed following vaccination of human volunteers was a major setback for HIV vaccine development. However, vaccine associated enhancement of virus replication had previously been described for experimental lentiviral vaccines targeting simian immunodeficiency virus, equine infectious anaemia virus, caprine arthritis encephalitis virus and FIV [47,48,49,50,51,52,53,54,55,56]. Thus, it is timely to increase our understanding of lentivirus vaccine-induced enhancement of infection, as this will be of pivotal importance to the development of successful lentiviral vaccines in the future.

Enhancement following vaccination against FIV was observed with a range of vaccine formulations [48,49,50,51,52,57,58], manifesting as increased viral loads and accelerated viraemia post-challenge. Antibody-dependent enhancement of infection has been well-documented in HIV and EIAV infections [47,59] and a comparison of X4 and R5 variants of HIV showed that monoclonal antibodies against gp120 which either neutralised or had a neutral effect against X4 variants, enhanced infection with R5 variants [60,61,62]. Thus, a humoral immune response that protects against an X4 strain of virus might enhance infectivity with an R5 strain of virus. By analogy, there is the potential that the current FIV vaccine might protect cats against challenge with isolates with a CRD2-independent phenotype, but lead to enhanced infection with CRD2-dependent isolates. The role of antibody in vaccine-mediated enhancement of FIV infection is unclear [57], but it is thought to be a contributory factor since the transfer of plasma between animals transferred enhancement of infection [51]. Recently, evidence from in vitro studies showed that plasma from cats infected with GL8 contains antibodies that neutralise homologous virus efficiently, but have a neutral effect or show enhancing activity against heterologous virus [63]. Addressing the phenomenon of enhancement will be crucial to the development of reliable and efficacious lentiviral vaccines.

## 10. Conclusions

The whole inactivated virus, dual-subtype FIV vaccine confers immunity to a range of heterologous strains, but does not protect cats against challenge with GL8. GL8 is a CRD2-dependent strain of FIV that is typical of the viruses that are transmitted in the field, since epidemiological evidence suggests that transmission is likely to occur from healthy cats with high viral burdens, i.e., before the switch to the CRD2-independent phenotype has occurred. Therefore, the next generation of FIV vaccines should be designed to boost immunity to CRD2-dependent strains. If our hypothesis that vaccinal immunity is related to receptor phenotype is confirmed, perhaps the affinity of the FIV-CD134 interaction might dictate the ability to escape from neutralising antibody and to turn a protective response into an enhancing one. Progress towards the development of lentiviral vaccines will be made, encouraged by the success as well as the known limitations of the FIV vaccine. Perhaps, if we can strengthen the antigen-specific immune response further whilst removing deleterious “enhancing” epitopes, it will be possible to protect cats from challenge with CRD2-dependent strains. Such an outcome would not only improve the prospects for vaccination of cats against FIV, but would encourage new strategies for the development of vaccines against the lentiviruses of humans and other species.
